# Acute and chronic changes in rat soleus muscle after high‐fat high‐sucrose diet

**DOI:** 10.14814/phy2.13270

**Published:** 2017-05-22

**Authors:** Kelsey H. Collins, David A. Hart, Ian C. Smith, Anthony M. Issler, Raylene A. Reimer, Ruth A. Seerattan, Jaqueline L. Rios, Walter Herzog

**Affiliations:** ^1^ Human Performance Laboratory University of Calgary Calgary Alberta Canada; ^2^ McCaig Institute for Bone and Joint Health University of Calgary Calgary Alberta Canada; ^3^ The Centre for Hip Health & Mobility Department of Family Practice University of British Columbia Vancouver British Columbia Canada; ^4^ Alberta Health Services Bone & Joint Health Strategic Clinical Network Calgary Alberta Canada; ^5^ Department of Mechanical Engineering University of Calgary Calgary Alberta Canada; ^6^ Department of Biochemistry and Molecular Biology University of Calgary Calgary Alberta Canada; ^7^ CAPES Foundation Brasilia Brazil

**Keywords:** Aerobic capacity, high‐fat/high‐sucrose diet, oxidative stress, rat model, soleus muscle

## Abstract

The effects of obesity on different musculoskeletal tissues are not well understood. The glycolytic quadriceps muscles are compromised with obesity, but due to its high oxidative capacity, the soleus muscle may be protected against obesity‐induced muscle damage. To determine the time–course relationship between a high‐fat/high‐sucrose (HFS) metabolic challenge and soleus muscle integrity, defined as intramuscular fat invasion, fibrosis and molecular alterations over six time points. Male Sprague‐Dawley rats were fed a HFS diet (*n* = 64) and killed at serial short‐term (3 days, 1 week, 2 weeks, 4 weeks) and long‐term (12 weeks, 28 weeks) time points. Chow‐fed controls (*n* = 21) were killed at 4, 12, and 28 weeks. At sacrifice, animals were weighed, body composition was calculated (DXA), and soleus muscles were harvested and flash‐frozen. Cytokine and adipokine mRNA levels for soleus muscles were assessed, using RT‐qPCR. Histological assessment of muscle fibrosis and intramuscular fat was conducted, CD68^+^ cell number was determined using immunohistochemistry, and fiber typing was assessed using myosin heavy chain protein analysis. HFS animals demonstrated significant increases in body fat by 1 week, and this increase in body fat was sustained through 28 weeks on the HFS diet. Short‐term time‐point soleus muscles demonstrated up‐regulated mRNA levels for inflammation, atrophy, and oxidative stress molecules. However, intramuscular fat, fibrosis, and CD68^+^ cell number were similar to their respective control group at all time points evaluated. Therefore, the oxidative capacity of the soleus may be protective against diet‐induced alterations to muscle integrity. Increasing oxidative capacity of muscles using aerobic exercise may be a beneficial strategy for mitigating obesity‐induced muscle damage, and its consequences.

## Introduction

Obesity is a global health epidemic, impacting musculoskeletal health and overall health, across the lifespan (Singh et al. 2016). Muscle is a key site for glucose regulation, and it is vulnerable to compromise both morphologically and biologically with obesity as it undergoes regular remodeling (Tidball 2005; Akhmedov and Berdeaux 2013). Using a rat model of high‐fat/high‐sucrose (HFS) diet induced obesity (DIO) across a six time points, we have demonstrated that HFS leads to increased intramuscular lipids, inflammatory cells, and tissue fibrosis in the vastus lateralis muscle of the quadriceps complex (Collins et al. 2016a), findings consistent with other high‐fat diet animals models (Fink et al. 2014). This compromise in muscle integrity can be observed at very early time points, as early as 3 days on HFS, and appears to be sustained in glycolytic skeletal muscles (Collins et al. 2016c).

Using a metabolic challenge, studies have indicated differential responses (i.e. disparate mitochondrial dysfunction) between primarily oxidative (e.g., rat soleus, >90% myosin heavy chain type I and IIA), and primarily glycolytic muscles (e.g., rat vastus lateralis, >90% myosin heavy chain type IIx and IIb fibers) (Rivero et al. 1999; Bloemberg and Quadrilatero 2012), such that primarily glycolytic muscles exhibit increased vulnerability to metabolic challenge, which may be at least partially due to limited oxidative capacity (Chanseaume et al. 2006; Matsakas and Patel 2009; Shortreed et al. 2009; Warren et al. 2014; Collins et al. 2016a). There is an opportunity to determine whether a protective mechanism exists within muscles of high aerobic capacity, with the rat soleus muscle being an example of such a muscle (Philippi and Sillau 1994). However, studies detailing short‐term and long‐term responses in oxidative soleus muscle integrity to metabolic challenge are lacking (Collins et al. 2016c). Although many previous studies aimed at producing muscle atrophy (denervation, casting, hind limb suspension) in the soleus muscle (Bodine et al. 2001), there remains a paucity of studies describing the soleus muscle in the context of diet‐induced obesity (Yu et al. 2008) across a spectrum of time points. By comparing soleus muscle integrity to previous findings in glycolytic muscles, a more comprehensive understanding of the effect of short‐term and long‐term metabolic challenge to muscle integrity can be generated, and used to inform studies aimed to develop appropriate exercise protocols.

The purpose of the present studies was to determine if previous results in the primarily glycolytic vastus lateralis are representative of all muscles, or whether a more oxidative muscle, as represented by the soleus, exhibits a different response to HFS Diet, indicated by the conservation of soleus muscle integrity. We assessed the soleus muscle across various time periods when subjected to a HFS metabolic challenge, using three primary outcome methods: histology, molecular biology, and myosin heavy chain protein analysis. We hypothesized that the structural integrity of the soleus would be preserved against the challenge of a high‐fat/high‐sucrose diet.

## Methods

Eighty‐five male Sprague‐Dawley rats were individually housed on a 12/h dark/light cycle, and were allocated to either a HFS diet group (39.2% of total energy as fat 43.5% sucrose, 15.8% protein; custom Diet #102412, Dyets, Inc, *n* = 64) or to a control chow‐diet (Chow, 13.4% of total energy as fat, 3.8% sucrose, 29.8% protein, *n* = 21, Lab Diet 5001). The energy density of the HFS diet was 4.6 kcal/g and 3.34 kcal/g for chow. The composition of these diets can be found elsewhere (Collins et al. 2015b). All experiments were approved by the University of Calgary Life and Environmental Sciences Animal Care Committee, and experiments were conducted in accordance with animal care standards. Animals were 10 ± 2 weeks old at the start of the *ad libitum* feeding intervention, and were allocated to one of the following groups: (a) killed after 3 days on HFS diet (*n* = 6), (b) killed after 1 week on HFS diet (*n* = 6), (c) killed after 2 weeks on HFS diet (*n* = 6), (d) killed after 4 weeks on HFS diet (*n* = 6) or to a short‐term (4 week) chow control‐diet group (*n* = 7). All short‐term animals were 15 ± 1.5 week old at the time of killing. Animal ages at the beginning and end of metabolic challenge, and age difference from chow at the end of metabolic challenge have been reported previously (Collins et al. 2016c). Forty HFS animals and 14 chow animals were followed for a subsequent 8 weeks, for a total 12‐week HFS standard obesity induction period. At the end of the obesity induction period, HFS animals were tertile stratified based on change in mass over time, and the top 33% were allocated to the obesity prone (DIO‐P, *n* = 13) group, while the bottom 33% were allocated to the obesity resistant group (DIO‐R, *n* = 13). The middle tertile were not further considered here. Approximately, half (*n* = 6 or 7) of the animals from each group were killed after the 12‐week obesity induction period, while the other half were monitored throughout a subsequent 16‐week adaption period (28‐week total), and then euthanized. Animals were killed by barbiturate overdose (Euthanyl^®^, MTC Animal Health Inc., Cambridge, Ontario, Canada). Immediately after killing, body composition was measured using Dual Energy X‐ray Absorptiometry and analyzed with software for small animal analysis (Hologic QDR 4500; Hologic, Bedford, MA). Blood serum was collected, prepared, and analyzed for glucose, and protein as previously described (Collins et al. 2015a, 2016b). Body composition, body mass, and serum profiles have been reported elsewhere (Collins et al. 2015a, 2016a, 2016b, 2016c).

### Fiber typing

Soleus muscles were harvested, weighed, and flash‐frozen in liquid nitrogen. Myosin heavy chain isoforms (MHC) from soleus muscle were separated using SDS‐page gel electrophoresis on 4.5% and 7.5% acrylamide stacking and separating gels, respectively, according to previously described methods (Joumaa et al. 2015), using a Bio‐Rad (USA) Mini‐Protean unit (73V for 40 h). Gels were stained with Coomassie Blue, and imaged on a GS‐800 Calibrated Densitometer (Bio‐Rad). Lane densities were quantified and the optical densities of the bands corresponding to MHC I, IIa, IIx, and IIb were determined, using the Gel Analysis features of Image J.

### Histological staining procedures

Briefly, a sample from the soleus mid belly of the tissue was cut, mounted in OCT compound, and 10 *μ*m sections were prepared, using a cryostat at −25°C, and then dried on slides at room temperature. Oil Red O (ORO) staining for fat, Picrosirius red staining for collagen, and CD68^+^ immunohistochemistry were completed as previously described, and detailed procedures can be found elsewhere (Collins et al. 2016a). ORO and Picrosirius red stained sections were then imaged at 10 ×  magnification and images were analyzed, using a custom MatLab program. The relative staining intensity for each animal was the average across the entire cross‐section of each muscle section (10–25 slides/animal). CD68^+^ cell counts were determined from the average cell counts measured in four randomly selected images taken across each muscle cross‐section at 20 × magnification (Olympus, Japan). Muscle circumference measurements were performed by quantifying the perimeter of each muscle, using a custom Matlab^®^ program.

### Tissue qPCR

Samples of mid‐belly frozen soleus muscle were processed as previously described using the Tri‐Spin method (Reno et al. 1997). Oxidative stress (iNOS), fibrosis (collagen I and III), oxidative capacity (succinate dehydrogenase, SDH), antioxidant scavenging (superoxide dismutase 2, SOD2) proinflammatory (IL‐1*β*, COX‐2, MCP‐1, TNF‐*α*, IL‐6, leptin), atrophy (MuRF‐1, MAFbx/atrogin‐1), and fat cell differentiation (PPARϒ) markers were evaluated using validated primers, as reported previously (Collins et al. 2016a,c). All assessments were performed in duplicate under optimal conditions that conformed to qPCR criteria.

### Statistical analysis

All groups were compared against their respective chow control diet group. Levene's test for equality of variance was conducted on all outcomes. If significant (*P* < 0.05), Kruskal‐Wallis nonparametric tests were used to evaluate each time point compared to control, followed by Dunn's Method of post hoc analysis if necessary (SigmaStat). If equal variances were found, subsequently, either ANOVA followed by Tukey's HSD post hoc analysis or Student's t‐tests for independent samples were performed between each individual HFS time point and the corresponding chow control group time point (IBM SPSS 21, *α *= 0.05).

## Results

### Muscle mass and morphology

**Figure 1:**
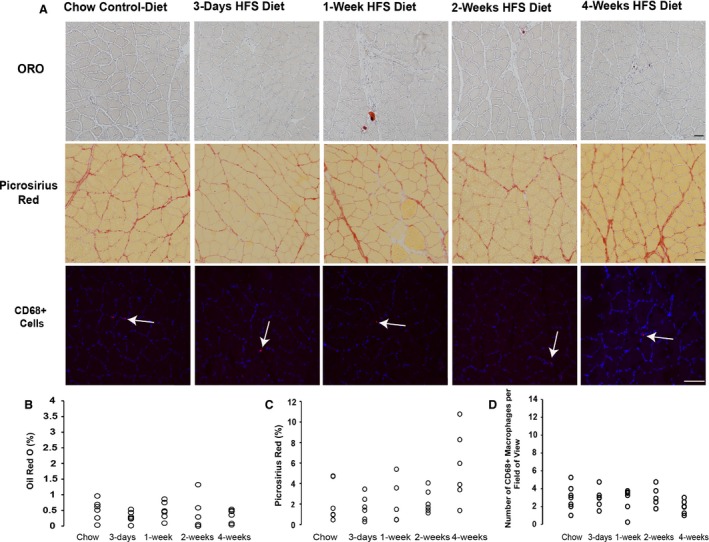
High‐fat high‐sucrose diet did not result in structural changes or increased CD68^+^ cell number in soleus muscle compared to chow‐fed control rats. (A) Top row: Oil Red O stainining for intramuscular lipid in soleus muscle sections taken at 100× magnification; middle row: Picrosirius red staining for collagen in soleus muscle sections, imaged at 100× magnification; bottom row: immunohistochemistry staining for CD68^+^ cells in soleus muscle sections, imaged at 200× magnification. White arrows demonstrate positive staining for CD68^+^ cells. (B) Raw average values for Oil Red O Staining for each animal, where 10–25 images were evaluated for a mid‐belly cross‐section. Black scale bar represents 100 *μ*m, images were taken at 100× magnification. (C) Raw average values for Picrosirius red staining for each animal, where 10–25 images were evaluated for a mid‐belly cross‐section. Black scale bar represents 100 *μ*m, images were taken at 100× magnification. (D) Raw average values for CD68^+^ staining for each animal, where 4 images were randomly selected and evaluated for a given mid‐belly cross‐section. White scale bars represent 100 *μ*m, images were taken at 200× magnification.

Muscle circumference and muscle mass measurements (Fig. S1) were conserved over time (*P* > 0.05), despite previously reported increases in body fat as early as 1 week on HFS compared to chow (Collins et al. 2016c). Soleus muscles from animals on HFS for 3 days, 1 week, and 2 weeks were similar to chow control diet animals. Soleus muscles exhibited similar levels of ORO staining and connective tissue content across the short‐term metabolic challenge as the soleus muscles from the chow control group animals (Fig.** 1). A trend toward increased connective tissue content was observed at 4 weeks (*P* = 0.078 vs. chow), but this did not progress over time.

At the end of the 12‐week obesity induction period, soleus fat content, connective tissue content, and CD68^+^ cell number, were similar across all groups, regardless of diet or obesity response (Table 1 *, P *>* *0.05). Differences in body mass and adiposity were retained with prolonged exposure to HFS in DIO‐P animals, while DIO‐R animals had higher body fat compared to chow control animals, but had similar body mass when compared to the corresponding control group animals, as previously reported (Collins et al. 2015a, 2016a, 2016b).

**Table 1:** Soleus Muscle Integrity is conserved across 12 and 28 weeks of high‐fat/high‐sucrose metabolic challenge compared to chow‐fed control animals

Group	12 weeks			28 weeks		
	Oil red O % (lipid)	Picrosirius red % (fibrosis)	CD68^+^ cells (per 0.15 mm^2^ field of view)	Oil red O % (lipid)	Picrosirius red % (fibrosis)	CD68^+^ cells (per 0.15 mm^2^ field of view)
DIO‐P	0.4 ± 0.1%	6.6 ± 0.5%	1.5 ± 0.2	0.3 ± 0.1%	8.0 ± 1.0%	3.1 ± 0.4
DIO‐R	0.2 ± 0.06%	7.6 ± 0.6%	1.5 ± 0.1	0.6 ± 0.3%	6.6 ± 1.0%	2.7 ± 0.8
Chow	0.2 ± 0.04%	5.9 ± 0.3%	1.3 ± 0.1	0.2 ± 0.1%	8.6 ± 1.0%	2.9 ± 0.2

DIO, diet induced obesity.

### Myosin heavy chain distribution

**Figure 2:**
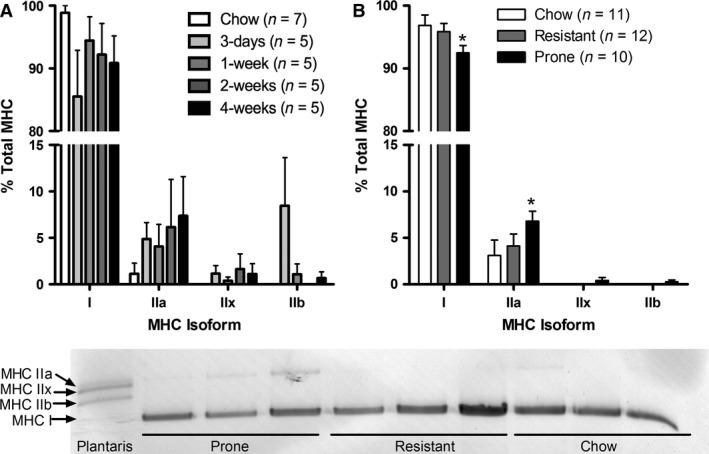
Myosin heavy chain (MHC) distribution was conserved in soleus muscles from chow‐fed control rats and high‐fat/high‐sucrose diets. (A) MHC distribution from animals on high‐fat/high‐sucrose (HFS) from 3 days to 4 weeks. (B), With tertile stratification into obesity prone and resistant groups as described in the methods (12 and 28 weeks pooled together obesity prone animals demonstrated a decreased proportion Myosin heavy chain (MHC) I, accompanied with an increase proportion of in MHC IIa. C) A sample gel is shown to demonstrate the separation between the four different MHC isoforms in chow‐fed control animals, obesity prone, and obesity resistant soleus.

The soleus muscles from all animals were predominantly composed (>98% on average) of MHC I and MHC IIa isoforms, which are associated with high oxidative potential. No significant differences in MHC distribution were detected in the first 4 weeks of HFS exposure, although there was a general trend toward less MHC I and more MHC IIa with HFS (Fig. 2A). Similarly, soleus muscles from obesity‐prone rats tended to have more MHC IIa and less MHC I than controls after 12 and 28 weeks on the HFS diet, although these differences were not significant when time points were examined in isolation (Fig. 2B). When the results from the 12‐ and 28‐week group animals were pooled, the soleus of obesity prone animals had proportionally less MHC I and more MHC IIa than the soleus of chow control group animals. The obesity‐resistant rats did not differ significantly from either the chow control group or the obesity‐prone group animals. None of the 18 soleus muscles assessed from the chow‐fed control rats had detectable levels of the MHC isoforms associated with low oxidative potential (MHC IIx and IIb). In contrast, 7 of the 42 soleus muscles assessed from the HFS rats at the various time points had detectable MHC IIx and/or IIb content, though no significant changes in muscle MHC IIx or MHC IIb composition were detected at any time point.

### Muscle RT‐qPCR

Despite similarities across groups in soleus ORO or picrosirius red staining (Fig. 1), marked increases in mRNA levels for some of the molecules assessed were observed (Table** 2). Specifically, mRNA levels for iNOS, were elevated across all short‐term time points in the soleus muscle of HFS group animals compared to chow group control animals (*P* < 0.05). Fluctuating, but significant increases in mRNA levels for oxidative stress scavenging (SOD2), oxidative capacity (SDH), pro‐inflammatory (COX‐2, TNF‐*α*, IL‐6), atrophy (MuRF‐1) and fat‐cell differentiation (PPARϒ) markers were observed across the short‐term time points, despite no detectable indications for altered structural integrity. Interestingly, MCP‐1 levels were not altered in soleus muscles with the short‐term metabolic challenge. In addition, there were no differences in mRNA levels for IL‐1*β*, collagen‐1, or collagen‐3 detected compared to chow group control animals (Table 2).

**Table 2:** Fluctuating, but significant changes in soleus muscle mRNA levels were observed, despite similarities in muscle structure with high‐fat/high‐sucrose diet

Marker type	Factor	3‐days fold‐change (SE)	1‐week mean fold‐change (SE)	2‐weeks fold‐change (SE)	4‐weeks fold‐change (SE)
Oxidative Stress	iNOS	21.5 ± 9.50^b^	23.80 ± 22.59^c^	74.00 ± 24.05^d^	38.68 ± 14.55^d^
Oxidative stress scavenger	SOD2	1.61 ± 0.25^b^	0.94 ± 0.07	3.0 ± 0.64^b^	2.28 ± 0.45^b^
Oxidative capacity	SDH	1.54 ± 0.40	1.06 ± 0.10	3.61 ± 0.71^b^	3.41 ± 0.64^b^
Pro‐inflammatory	COX‐2	125.22 ± 55.72^d^	121.36 ± 120.35	419.53 ± 153.26^d^	209.50 ± 73.27^c^
	IL‐6	89.43 ± 38.06^d^	15.50 ± 14.58	400.19 ± 100.13^c^	197.59 ± 76.65^b^
	Leptin	1.49 ± 0.82	0.55 ± 0.11	9.95 ± 5.40^a^	2.41 ± 1.59
	MCP‐1	1.27 ± 1.22	0.71 ± 0.04	0.50 ± 0.09	0.64 ± 0.11
	TNF‐*α*	1.93 ± 0.30^b^	1.31 ± 0.43	2.55 ± 0.39^d^	2.03 ± 0.85
Fat cell differentiation	PPARϒ	2.16 ± 0.52^c^	1.16 ± 0.08	3.59 ± 0.52^c^	2.51 ± 0.72^c^
Atrophy	MuRF‐1	1.81 ± 0.47^b^	2.76 ± 1.75	3.54 ± 1.79^b^	2.75 ± 0.56^b^
	MAFbx/atrogin‐1	1.18 ± 0.09	1.42 ± 0.56	1.99 ± 0.57^b^	2.15 ± 0.57^b^

No significant changes in mRNA levels for any markers were observed at 12 or 28 weeks of diet exposure.

a Indicates *P* < 0.10 versus control.

b Indicates *P* < 0.05 versus control.

c indicates *P* < 0.01 versus control.

d *P* < 0.001 versus control.

After 12 weeks on HFS, SOD2 was significantly decreased in DIO‐P and DIO‐R animals compared to chow, but there was a trend toward increased SOD2 mRNA levels at 28 weeks (Table S1). Furthermore, TNF‐ *α* levels were significantly increased in DIO‐P compared to chow at 28 weeks. No other significant differences were detected in mRNA levels for any of the molecules evaluated after 12 and 28 weeks of obesity induction across all markers evaluated.

## Discussion

The present studies demonstrate that soleus muscle structural integrity is essentially conserved over short‐ and long‐term exposure times to a HFS diet, despite previous reports of low‐level systemic inflammation, as indicated by serum inflammatory profiles, and metabolic perturbations in other muscles of these animals (Collins et al. 2015a, 2016c). For instance, very early and potentially deleterious changes in muscle inflammation and structural integrity have been reported following short‐term exposure to a HFS diet in the vastus lateralis (VL), a glycolytic muscle (Collins et al. 2016a). In rat VL, these were manifested by a host of systemic inflammatory changes (Fink et al. 2014; Collins et al. 2016a). Thus, it appears that a protective mechanism may be operative to preserve the structure of the largely oxidative soleus muscle in animals exposed to a HFS diet, especially compared to muscles with a glycolytic fiber type (Coen et al. 2013; Banse et al. 2015; Collins et al. 2016a,c; Hyatt et al. 2016).

Oxidative stress has been shown to impair the oxidative capacity of muscles, whereby glycolytic muscles, such as the VL, may be more vulnerable to damage due to their inherently lower oxidative capacity (Schrauwen and Hesselink 2004; Yokota et al. 2013; Warren et al. 2014). In a study of nonhuman primates, a type I to type II MHC switch was observed with a HFS diet, but this shift was blunted with dietary resveratrol supplementation (enhancing expression of PCG‐1*α* and mitochondrial biogenesis) (Hyatt et al. 2016). These primate findings are parallel those demonstrated here in the rat (Collins et al. 2016a), and in other rodent populations (Akhmedov and Berdeaux 2013; Fink et al. 2014). Despite similarities across groups in soleus ORO or picrosirius red staining, we observed a small but significant slow to fast MHC isoform shift in HFS soleus muscles at 28 weeks, similar to previous reports (reviewed in Matsakas and Patel 2009). While the impact of this myosin isoform shift on the oxidative capacity of the soleus is unknown, it is possible that the increase in MHC IIa represents an adaptive strategy to increase muscle oxidative capacity as fibers expressing MHC IIa have the highest SDH activity (a marker of oxidative capacity) in a wide range of muscles in the rat (Rivero et al. 1999; Bloemberg and Quadrilatero 2012). Recent evidence demonstrates that 12 weeks of high‐fat or high‐sucrose feeding impairs mitochondrial function in glycolytic quadriceps muscles of rats (Jørgensen et al. 2017). In the present studies, SDH mRNA levels were increased after 2 and 4 weeks of HFS diet, suggesting a short‐term compensatory increase in oxidative capacity with metabolic disturbance. After 12 weeks of HFS diet, however, mRNA levels for SDH were similar between DIO and chow‐fed diet, supporting the notion that early adaptation may result in the compensatory shift (i.e MHC I to IIa) observed over longer exposures to HFS diet. If this speculation is correct, it could be considered corroborating evidence that high oxidative capacity is a protective mechanism mitigating the deleterious effects associated with obesity‐inducing diets, and future studies will validate these mRNA findings with direct measurements of SDH activity in this context.

Previous research suggests that oxidative capacity increases with high‐fat diets in an attempt to mitigate the associated metabolic insult, further linking oxidative capacity and oxidative stress with obesity and related complications (Matsakas and Patel 2009; Savini et al. 2013). In fact, after 12 weeks, COX‐2 mediated events may be related to the induction of insulin resistance in a model of high‐fat diet‐induced obesity (Tian et al. 2011). Previous authors observed an increase in COX‐2 and subsequent insulin resistance in adipose tissue and glycolytic muscles, but not the rat soleus muscle (Tian et al. 2011). In agreement with this previous study, we did not observe increases in COX‐2 at 12 weeks, but the dynamic increases in mRNA levels for several markers (including COX‐2, as well as the atrophy markers MuRF‐1 and MAFbx/atrogin‐1, etc), that were observed at the very early time points evaluated (3 days, 2 weeks, 4 weeks) may indicate a perturbation that the soleus muscle is managing, and ultimately adapting to, to prevent damage and conserve integrity.

Increased capacity for cellular antioxidant defense may be another reason the soleus muscle is protected with long‐term metabolic challenge. In situ, glycolytic fibers demonstrate decreased H_2_O_2_ scavenging capacity, measured by decreases in SOD2, which is an indicator of superoxide formation, when compared to oxidative fibers from the soleus muscle (Anderson and Neufer 2005). However, glutathione peroxidase, considered responsible for H_2_O_2_ removal, is reported to be similar across muscles regardless of fiber type (Anderson and Neufer 2005). These data suggests that reduced oxidative stress scavenging, rather than oxidative stress removal, is one source of potential vulnerability in glycolytic muscles with metabolic challenge (Anderson and Neufer 2005). Furthermore, mice with increased SOD2 expression are protected from insulin resistance with diet‐induced obesity (Hoehn et al. 2009), and demonstrate increased lipid oxidation in skeletal muscle and adipose tissue (Liu et al. 2013), preventing oxidative damage in the muscle. In the present study, soleus muscles from DIO rats show an initial dynamic increase in mRNA levels for SOD‐2 at 3 days, 2 weeks and 4 weeks compared to chow. This suggests an intramuscular effort at employing increased antioxidant defense with HFS challenge. After 12 weeks of HFS diet, however, decreased mRNA levels for SOD2 were measured in DIO animals compared to chow‐fed controls, which is concordant with previous work indicating decreased expression of antioxidant proteins in humans with obesity (Tinahones et al. 2009). However, this decrease in SOD2 may be transient, as a trend toward an increase in SOD2 in DIO animals compared to chow was observed by 28 weeks, suggesting that the soleus muscle may again compensate over time. Despite this, no structural damage is evident in the soleus muscles identified, suggesting there are likely redundant mechanisms for managing the oxidative stress induced by metabolic dysfunction.

Levels of mRNA for MuRF‐1 and MAFbx/atrogin‐1, two key markers for muscle atrophy, were increased in the absence of detectable structural damage. Although MuRF‐1 and MAFbx/atrogin‐1 are generally up or down‐regulated concordantly (Bodine and Baehr 2014), we observed a fluctuating increase in MuRF‐1 mRNA levels as early as 3 days following onset of the HFS diet, while those for MAFbx/atrogin‐1 were similar between HFS and chow animals until after 2 weeks on the HFS diet – further underscoring the dynamic perturbation from short‐term exposure to a HFS diet. Moreover, TNF‐*α* has a role in muscle loss with obesity (Spiegelman and Hotamisligil 1993; Sishi et al. 2011), and it is reported to induce MAFbx/atrogin1 signaling through FOXO‐4 and p38 MAPK expression (Li et al. 2005; Moylan et al. 2008). P38 MAPK, in turn, is known to regulate iNOS, COX‐2, and MAFbx/atrogin‐1. It is interesting that despite considerable increases in the mRNA levels for these markers in the soleus, on average, muscle integrity was largely unchanged, even after long periods of exposure (Sishi et al. 2011; Yang et al. 2014). Despite the increases in MAFbx/atrogin‐1, nitric oxide antioxidant defenses have been demonstrated to block TNF‐*α* induced MAFbx/atrogin‐1 mRNA expression (Yu et al. 2008)*,* which may explain, in part, increases in MAFbx/atrogin‐1 mRNA levels observed without changes in soleus muscle integrity, and may be one mechanism by which soleus muscle integrity is conserved following exposure to a HFS diet.

This study provides insight into the manner by which obesity affects individual muscles during disease onset, which is a central element to our understanding of the appropriateness and impact of aerobic exercise in managing, treating, or preventing obesity. These results suggest a role for increasing aerobic capacity, perhaps by aerobic exercise, in preserving, mitigating, or preventing risks to muscle integrity in individuals with obesity, specifically in glycolytic muscles (Short et al. 2003; Louche et al. 2013). In humans, low aerobic capacity is a predictor of cardiovascular disease and all cause‐mortality independent of activity (Kodama et al. 2009; Kokkinos et al. 2010). Using an animal model of high‐ and low‐capacity running, or equivalently, high‐ and low‐aerobic capacity (Garton et al. 2016; Thyfault and Wright 2016), previous studies demonstrated that both maintaining high oxidative capacity and improving oxidative capacity via exercise protects against structural and functional consequences due to high‐fat diet induced metabolic challenge. Mice that are selectively bred for high aerobic capacity demonstrate less intramuscular lipid in the medial gastrocnemius when given a high‐fat diet compared to low‐aerobic capacity animals (Noland et al. 2007). This highlights a role for innate oxidative capacity in blocking or preventing obesity‐induced loss of muscle integrity. In addition, compensatory increases in spontaneous physical activity, and preservation of normal muscle metabolic function, with maintenance of muscle adenosine monophosphate‐activated protein kinase (AMPK) activity, may protect the high aerobic capacity animals from impaired insulin resistance (Park et al. 2016). Future work will determine an oxidative threshold for muscle damage beyond endogenous resistance or protection against a metabolic threat to understand vulnerability in glycolytic muscles. As such, interventions centered on increasing muscle oxidative capacity and reducing low‐level systemic inflammation will be developed and tested in this model system as potentially viable strategies to mitigate early damage due to a HFS diet.

## Conflict of Interest

None declared.

## Supporting information




**Figure S1:** Soleus absolute muscle mass (A) and muscle mass relative to body mass (B) was similar between high‐fat/high‐sucrose‐fed and chow‐fed rats at each time‐point evaluated.

**Table S1:** superoxide dismutase 2 (SOD‐2) oxidative stress scavenger mRNA levels are down‐regulated in obesity prone and obesity resistant animals at 12 weeks, while other markers are similar between high‐fat/high‐sucrose diet and control animals.
