# Supplementary material for: Acute and chronic changes in rat soleus muscle after high‐fat high‐sucrose diet
Source: Physiol Rep. 2017 May 22;5(10):e13270. doi: 10.14814/phy2.13270 (PMC5449557; doi:10.14814/phy2.13270)
Supplement: Supplementary file 1 — Figure S1: Soleus absolute muscle mass (A) and muscle mass relative to body mass (B) was similar between high‐fat/high‐sucrose‐fed and chow‐fed rats at each time‐point evaluated. [file PHY2-5-e13270-s001.docx]

Supplementary Figure

A:

B:

**Supplementary Figure: Soleus absolute muscle mass (A) and muscle mass relative to body mass (B) was similar between high-fat/high-sucrose-fed and chow-fed rats at each time-point evaluated.**
