# Supplementary material for: Acute and chronic changes in rat soleus muscle after high‐fat high‐sucrose diet
Source: Physiol Rep. 2017 May 22;5(10):e13270. doi: 10.14814/phy2.13270 (PMC5449557; doi:10.14814/phy2.13270)
Supplement: Supplementary file 2 — Table S1: superoxide dismutase 2 (SOD‐2) oxidative stress scavenger mRNA levels are down‐regulated in obesity prone and obesity resistant animals at 12 weeks, while other markers are similar between high‐fat/high‐sucrose diet and control animals. [file PHY2-5-e13270-s002.docx]

**Supplementary *Table:*SOD-2 oxidative stress scavenger mRNA levels are down-regulated in obesity prone and obesity resistant animals at 12-weeks, while other markers are similar between high-fat/high-sucrose diet and control animals.**Despite increases in TNF-**α** in DIO-P vs. control at 28-weeks, muscle structure was not deleteriously altered with high-fat/high-sucrose diet. No other differences in mRNA levels were observed at 28-weeks of diet exposure.

|  |  | **12-weeks** | | **28-weeks** | |
| --- | --- | --- | --- | --- | --- |
| **Marker Type** | **Factor** | **DIO-P Mean**  **Fold-change (SE)** | **DIO-R Mean**  **Fold-change (SE)** | **DIO-P Mean**  **Fold-change (SE)** | **DIO-R Mean**  **Fold-change (SE)** |
| Oxidative Stress | **iNOS** | 0.76 ± 0.11 | 0.75 ± 0.17 | 0.99 ± 0.12 | 0.82 ± 0.19 |
| Oxidative Stress Scavenger | **SOD2** | **0.56 ± 0.10**** | **0.47 ± 0.06**** | 2.41 ± 0.35‡ | 2.01 ± 0.47‡ |
| Oxidative Capacity | **SDH** | 0.79 ± 0.10 | 0.64 ± 0.06 | 1.26 ± 0.13 | 1.02 ± 0.14 |
|  | **COX-2** | 0.71 ± 0.31 | 0.25 ± 0.014 | 1.02 ± 0.22 | 1.10 ± 0.15 |
|  | **IL-6** | 0.67 ± 0.29 | 0.71 ± 0.13 | 3.49 ± 0.50 | 2.81 ± 0.81 |
| Pro- | **Leptin** | 0.81 ± 0.24 | 0.65 ± 0.16 | 0.87 ± 0.27 | 0.92 ± 0.21 |
| Inflammatory | **MCP-1** | 0.97 ± 0.19 | 0.97 ± 0.27 | 1.14 ± 0.25 | 0.60 ± 0.19 |
|  | **TNF-α** | 1.19 ± 0.50 | 0.51 ± 0.08 | **1.91 ± 0.13*** | 1.35 ± 0.38 |
| Fat cell  differentiation | **PPARϒ** | 0.97 ± 0.17 | 0.78 ± 0.11 | 1.03 ± 0.18 | 0.80 ± 0.12 |
|  | **MuRF-1** | 0.99 ± 0.13 | 1.13 ± 0.12 | 1.03 ± 0.13 | 1.25 ± 0.23 |
| Atrophy | **MAFbx/atrogin-** | 0.86 ± 0.06 | 0.99 ± 0.17 | 0.99 ± 0.15 | 0.98 ± 0.21 |

‡ indicates p <0.10vs. control;

* indicates p<0.05vs. control;

** indicates p<0.01vs. control;
